# The Proto-Oncogene TWIST1 Is Regulated by MicroRNAs

**DOI:** 10.1371/journal.pone.0066070

**Published:** 2013-05-31

**Authors:** Maarja-Liisa Nairismägi, Annette Füchtbauer, Rodrigo Labouriau, Jesper Bertram Bramsen, Ernst-Martin Füchtbauer

**Affiliations:** Department of Molecular Biology and Genetics, Aarhus University, Aarhus, Denmark; The John Curtin School of Medical Research, Australia

## Abstract

Upregulation of the proto-oncogene *Twist1* is highly correlated with acquired drug resistance and poor prognosis in human cancers. Altered expression of this multifunctional transcription factor is also associated with inherited skeletal malformations. The mammalian *Twist1* 3′UTRs are highly conserved and contain a number of potential regulatory elements including miRNA target sites. We analyzed the translational regulation of TWIST1 using luciferase reporter assays in a variety of cell lines. Among several miRNAs tested, miR-145a-5p, miR-151-5p and a combination of miR-145a-5p + miR-151-5p and miR-151-5p + miR-337-3p were able to significantly repress *Twist1* translation. This phenomena was confirmed with both exogenous and endogenous miRNAs and was dependent on the presence of the predicted target sites in the 3′UTR. Furthermore, the repression was sensitive to LNA-modified miRNA antagonists and resulted in decreased migratory potential of murine embryonic fibroblast cells. Understanding the *in vivo* mechanisms of this oncogene's regulation might open up a possibility for therapeutic interference by gene specific cancer therapies.

## Introduction

The evolutionary conserved basic helix-loop-helix (bHLH) transcription factor TWIST1 is a multifunctional proto-oncogene with a strong correlation to poor prognosis. TWIST1 is able to inhibit c-MYC induced apoptosis [1] and directly regulates the expression of several other oncogenes such as GLI1 [2], miR-10b [3] and AKT2 [4]. Overexpression of *TWIST1* has been observed in various types of cancer such as breast, prostate, gastric, pancreatic and bladder cancer, hepatocarcinoma, rhabdomyosarcoma, and glioma and is often associated with more aggressive phenotypes, and acquired drug resistance (reviewed in [5]).

*Twist1* expression is regulated by a complex network of signals and has been described as an integrator of SHH, FGF and BMP signaling [6]. In mice, a genomic fragment containing 6 kb upstream and 1.5 kb downstream of the *Twist1* gene is not sufficient to recapitulate Twist1 expression during embryogenesis (unpublished data) which is consistent with the observation that a translocation breakpoint 3 kb downstream of the *Twist1* gene creates a comparable haploid insufficiency as the null allele [7]. However, a number of transcription factor binding sites have been identified in the Twist1 upstream region and direct binding of transcriptional activators like NF-κB [8] and repressors like Prospero-related homeobox 1 (PROX1) [9] has been shown.

*TWIST1* is directly upregulated by hypoxia-inducible factor-1α (HIF-1α) and HIF-2α [10], [11]. Intratumoral hypoxia is correlated with radiation therapy resistance and enhanced metastatic potential [12]. TWIST1 promotes tumor metastasis [10] by epithelial-mesenchymal transition (EMT) [13] and formation of invadopodia, the specialized membrane protrusions for extracellular matrix degradation [14]. TWIST1 is linked to the transcription factor SNAIL, which also induces EMT, in a positive feedback loop [15]. In addition, co-expression of *TWIST1*, *HIF-1α* and *SNAIL* has been correlated with metastasis and poor prognosis in primary tumors of head and neck squamous cell carcinoma (HNSCC) patients [10].

The wide spectrum of TWIST1 functions might be explained by the fact that TWIST1 is able to act both as a transcriptional activator [16] and inhibitor [17], [18].

Originally, the *Twist* gene was discovered as a mutation disturbing cellular motility and EMT during gastrulation in *Drosophila melanogaster* [19], [20]. A comparable phenotype is observed in murine homozygous *Twist1* null mutant embryos in which the cephalic neural crest cells are unable to form a functional mesenchyme [21]. Haploid insufficiency causes Saethre-Chotzen syndrome (polysyndactyly and craniosynostosis) in humans and comparable symptoms in mice [7], [22].

During mammalian embryogenesis, *Twist1* mRNA precedes TWIST1 protein expression, indicating a translational control of *Twist1* [23], [24]. The same phenomena was recently observed in MCF-10ANeoT cells that had undergone EMT [25]. It is therefore interesting that many of the biological processes in which TWIST1 is involved, are associated with regulation by miRNAs, a hallmark of translational control. MiRNAs are endogenous small non-coding RNAs that regulate gene expression post-transcriptionally by modulating mRNA translation or stability. They influence cellular processes such as EMT, apoptosis, and differentiation, which all are essential for development and cancer [26], [27]. MiRNA-mediated regulation of gene expression is complex as one mRNA is usually targeted by several miRNAs and one miRNA can target several mRNAs. Furthermore, miRNAs can act synergistically leading to higher levels of repression [28].

Here we show that the 3′UTRs of mammalian *Twist1* genes contain conserved miRNA target sites, which make them sensitive to regulation by several miRNAs, individually and in cooperative combination. Understanding the exact mechanism of *Twist1* regulation is important as it may allow to utilize this physiological process to be utilized therapeutically.

## Results

### Analysis of Twist1 3′UTR

Translational regulation of mRNAs is typically mediated through evolutionary conserved regulatory regions within the UTRs. To test if this is the case for *Twist1*, we compared the conservation of coding sequence (CDS) and 3′UTR of *Twist1* mRNA among selected amniotes. 5′UTRs were not included since the full-length sequences are not available for all species. Limiting the investigation to amniotes gave us the possibility to also compare the conservation of *Twist1* with that of *Twist2* (Table 1), a highly related gene that is differently expressed but functionally largely equivalent to *Twist1* [29]–[31].

**Table 1:** Pairwise comparison of *Twist1* and *Twist2* mRNA sequence domains of three selected amniotes with the corresponding human sequence. Numbers represent% sequence identity.

	*Twist1* CDS^1^	*Twist1* 3′UTR^2^	*Twist2* CDS^1^	*Twist2* 3′UTR^2^
**Human**	100	100	100	100
**Cow**	95	92	98	68
**Mouse**	93	87	97	65
**Chicken**	84	54	80	32

1 CDS: coding sequence; ^2^UTR: untranslated region.

**Figure 1:**
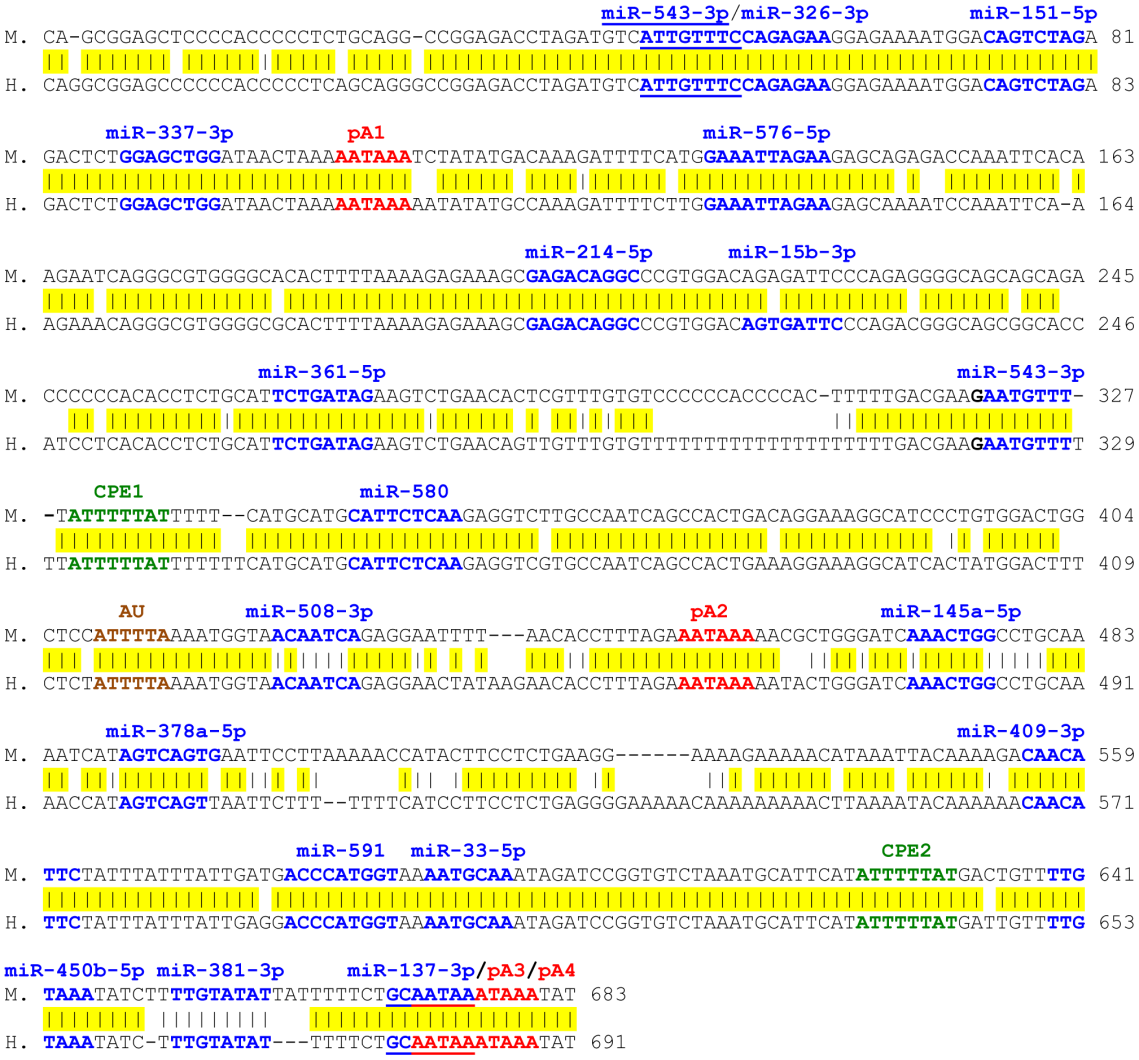
Alignment of human and murine *Twist1* 3′UTR sequences. Yellow represents the conserved nucleotides in the 3′UTR of mouse (upper), human (lower), cow, pig and dog *Twist1*. Polyadenylation signals are indicated in red, CPEs in green, AU-rich sequence in brown and potential miRNA target sites in blue. In overlapping sequences, potential miRNA target sites are individualized by underlining. AU: AU-rich sequence, CPE: cytoplasmic polyadenylation element, H.: *Homo sapiens*, M.: *Mus musculus*, pA: nuclear polyadenylation signal.

The two genes are highly similar in their coding regions, genomic structure and length of the 3′UTR. However, the 3′UTR of *Twist2* is not related to that of *Twist1* (percent of identity 30%) and is, between species, remarkably less conserved compared to the unusually highly conserved 3′UTR of *Twist1* indicating a functional selection of this sequence that is almost as stringent as for the CDS. Furthermore, the 3′UTR of *Twist1* contains considerably more potential regulatory sequences, namely four nuclear polyadenylation signals (pA1-4 where pA3 and pA4 overlap), two cytoplasmic polyadenylation elements (CPEs), one AU-rich sequence and a number of putative miRNA target sites predicted by several algorithms (TargetScan, miRBase and PicTar). All but one of the identified potential regulatory sequences (miR-15b-3p) are 100% conserved between mouse and human *Twist1* 3′UTR sequences (Fig. 1) and a large number are conserved in a wide range of mammalian species (Table 2). In addition, out of 18 miRNAs predicted to target *Twist1*, only one (miR-145a-5p) putatively targets *Twist2* as well (Table 2).

**Table 2:** Conservation of microRNA target sites in selected amniotes and the presence in *Twist2* 3′UTR.

MiRNA target site/Species	Human	Mouse	Cow	Dog	Chicken	Frog	Targeting *Twist2*
miR-15b-3p	+	−	+	+	−	−	−
miR-33-5p	+	+	+	+	−	+	−
miR-137-3p	+	+	+	+	−	+	−
miR-145a-5p	+	+	+	+	−	−	+
miR-151-5p	+	+	+	+	−	+	−
miR-214-5p	+	+	+	+	−	−	−
miR-326-3p	+	+	+	+	−	−	−
miR-337-3p	+	+	+	+	−	+	−
miR-361-5p	+	+	+	+	−	−	−
miR-378a-5p	+	+	+	+	−	−	−
miR-381-3p	+	+	+	+	−	+	−
miR-409-3p	+	+	+	+	−	−	−
miR-450b-5p	+	+	+	+	−	+	−
miR-508-3p	+	+	+	+	−	−	−
miR-543-3p	+	+	+	+	−	−	−
miR-576-5p	+	+	+	+	−	−	−
miR-580	+	+	+	+	−	−	−
miR-591	+	+	+	+	−	−	−

MicroRNAs underlined were tested in this study.

### MicroRNAs are targeting the Twist1 3′UTR

The presence of multiple potential miRNA target sites in the 3′UTR of *Twist1* led us to investigate whether *Twist1* expression could be regulated by miRNAs. The following miRNAs were tested for their potential to repress *Twist1* translation in the human lung carcinoma cell line H1299: miR-33, miR-145a, miR-151, miR-326, miR-337, miR-361, miR-378a, miR-381, miR-409 and miR-543 (Fig. 1).

**Figure 2:**
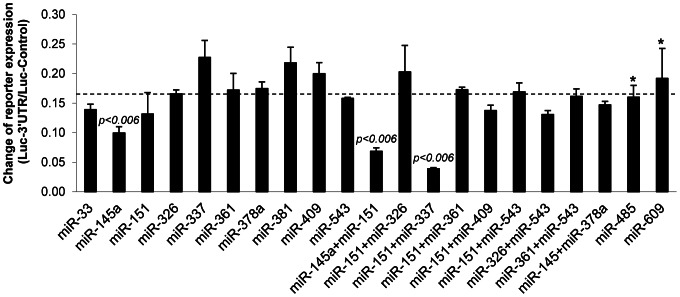
MicroRNAs repress the *Twist1* 3′UTR reporter. H1299 cells were transfected with the indicated miRNA, pRluc-N2 and pGL3-Twist1-3′UTR Firefly luciferase reporter or the empty pGL3-control vector and analyzed after 48 h. Firefly luciferase activities were normalized to the *Renilla* luciferase activities which served as internal standards, averages of triplicates were calculated and results were normalized to empty pGL3-control vector. The dashed line indicates the unrepressed expression level of the reporter (0,176; calculated from the average of two negative controls (*), miR-485 and miR-609). Statistical significance of miRNA effects was calculated by comparison with this average using Student's t-test.

Murine pre-miRNA sequences were cloned into pdCMV2-EGFP expression vector and their correct processing was confirmed by Northern Blot (not shown). To determine whether any of the selected miRNAs are able to repress the expression of TWIST1, the miRNAs were tested individually or in pairwise combinations in a luciferase reporter assay using a construct in which the Firefly luciferase CDS was followed by the murine *Twist1* 3′UTR sequence. MiR-485 and miR-609 have no target site in the 3′UTR of *Twist1* and were used as negative (mock) controls to calculate the unrepressed expression level of the reporter in these cells (Fig. 2). Expression of miR-145a resulted in a significant downregulation of the reporter by 44% (*p*<0.006). In addition, the co-expression of two pairs of miRNAs also led to a significantly increased repression: miR-151 + miR-337 resulted in a synergistic inhibition of 78% and miR-145a + miR-151 repressed TWIST1 expression by 61% (*p*<0.006). Notably, miR-337 alone had no effect while miR-151 only had a weak effect which in this assay was not statistically significant (*p*<0.15; see discussion).

**Figure 3:**
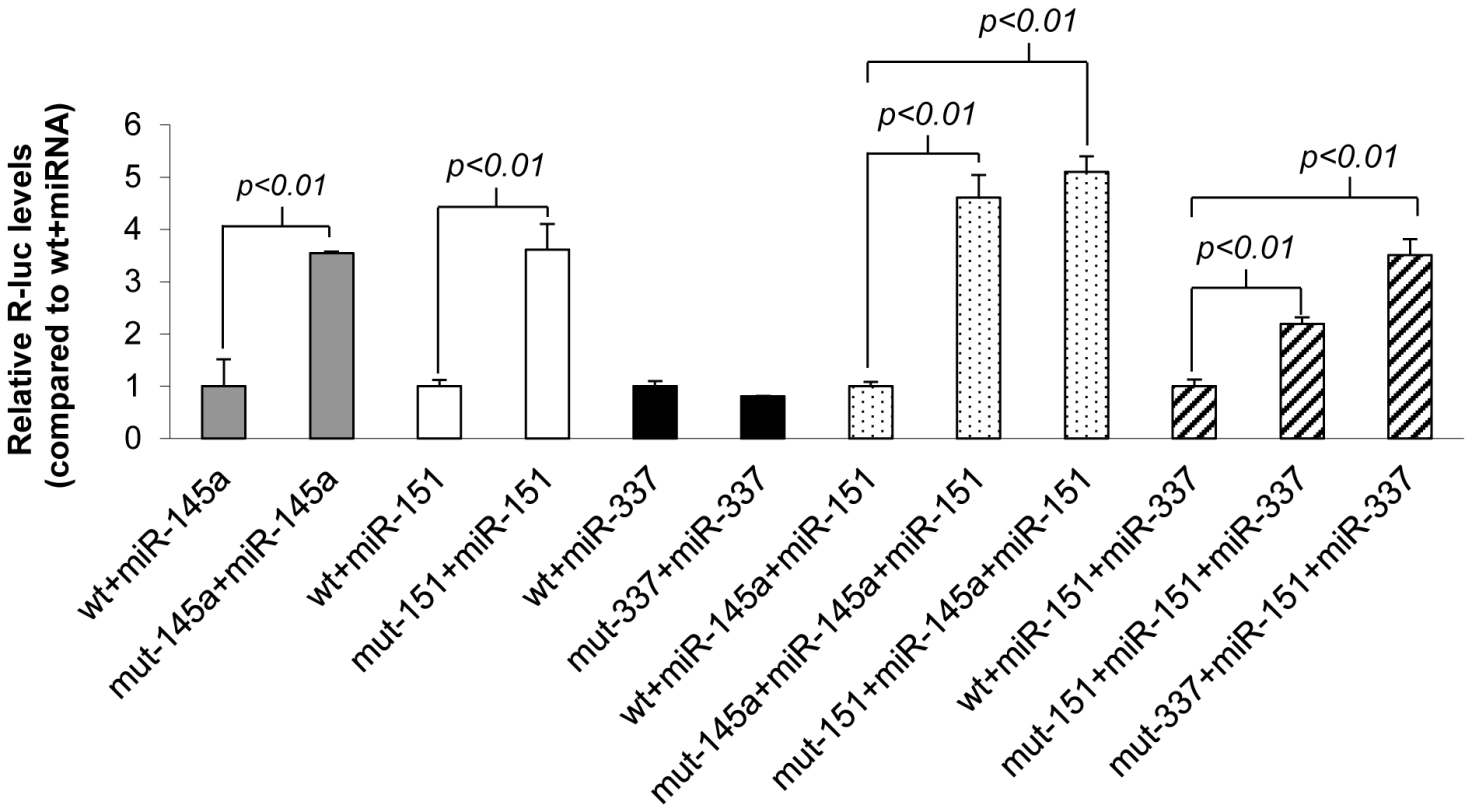
MicroRNA target sites are necessary for the repression of *Twist1* 3′UTR reporter. MiRNAs of interest were co-transfected with either wt or the corresponding mutant of psiCheck2-Twist1-3′UTR reporter into H1299 cells. Firefly luciferase activities were measured after 48 h, normalized to *Renilla* luciferase activities and subsequently to the respective wt+miRNA. Statistical significance was calculated by using Student's t-test and by comparing the mutant 3′UTR values with the wt 3′UTR reporter.

In order to confirm the location and functionality of the predicted miRNA target sites within the 3′UTR of *Twist1*, we mutated some of them to restriction enzyme recognition sites. Transfection of the wild type (wt) or mutant reporter with the corresponding miRNA confirmed that the repressive effects depended on the presence of the miRNA target sites. As shown in Fig. 3, mutating the target site of miR-145a-5p or miR-151-5p resulted in a loss of repression whereas lack of a binding site for miR-337-3p had no effect. When both miR-145a and miR-151 were present, mutating either of the target sites caused a comparable loss of repression. Interestingly, miR-337 was only able to repress the 3′UTR reporter when miR-151 and its target site both were present. This confirms that miRNAs and their target sites both are necessary to repress the expression of TWIST1 protein.

### MicroRNAs can act by translational inhibition

**Figure 4:**
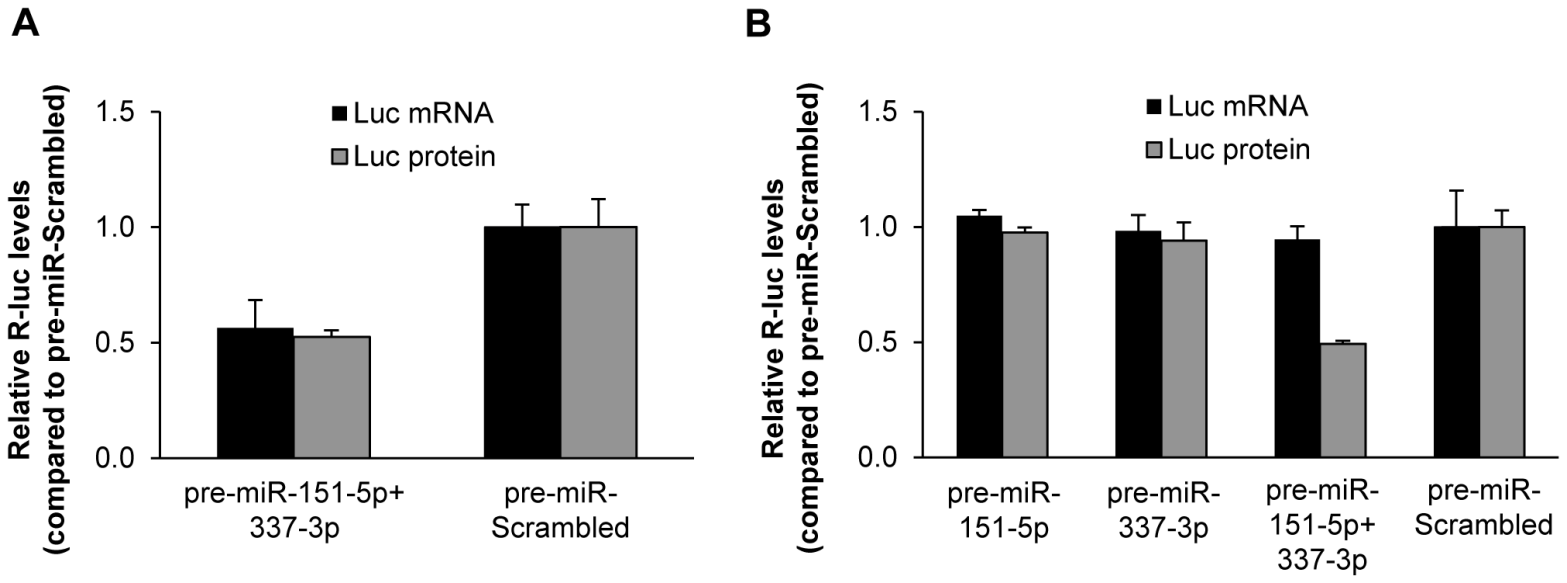
MicroRNAs lead to translational inhibition of *Twist1* 3′UTR reporter. (A) H1299 cells were co-transfected with wt psiCheck2-Twist1-3′UTR reporter and 20 nM synthetic precursor miRNAs. (B) H1299 cells stably expressing the psiCheck2-Twist1-3′UTR reporter were transfected with 5 nM synthetic precursor miRNAs. *Renilla* luciferase activity was measured after 48 h and normalized to the Firefly luciferase activity. *Renilla* luciferase mRNA levels were measured by qPCR, normalized to Firefly luciferase mRNA levels and subsequently to cells transfected with the negative control.

MicroRNAs primarily act by destabilizing target mRNA through decapping and/or deadenylation. However, a smaller subset of targets is rather translationally repressed leaving the mRNA levels unaltered (reviewed in [32]). As the uneven ratio between *Twist1* mRNA and TWIST1 protein in the mouse embryo [23], [24] indicated translational inhibition, we compared the mRNA and protein ratios in cells treated with a combination of synthetic miR-151-5p and miR-337-3p precursors. Using high concentrations of pre-miR-151-5p and pre-miR-337-3p (above 10 nM) we consistently found comparable decrease in both the mRNA and protein level of the *Twist1* 3′UTR reporter (Fig. 4A). To more closely mimic the physiological conditions, we established stable pools of cells harboring the *Twist1* 3′UTR reporter and transfected them using low concentrations (5 nM) of synthetic miR-151-5p and miR-337-3p precursors. Under such conditions, we observed a 50% reduction of luciferase reporter activity and hardly any reduction in the corresponding mRNA level upon miRNA co-transfection (Fig. 4B). This indicates that the synergistic effect of miR-151-5p and miR-337-3p is mainly due to translational inhibition and not RNA degradation. A corresponding analysis using the endogenous TWIST1 protein was technically not possible as we found all available TWIST1 specific antibodies to also react with unidentified proteins in murine cells not expressing TWIST1, a specificity issue not seen in human cells [25].

### Twist1 reporter is downregulated by endogenous microRNAs

**Figure 5:**
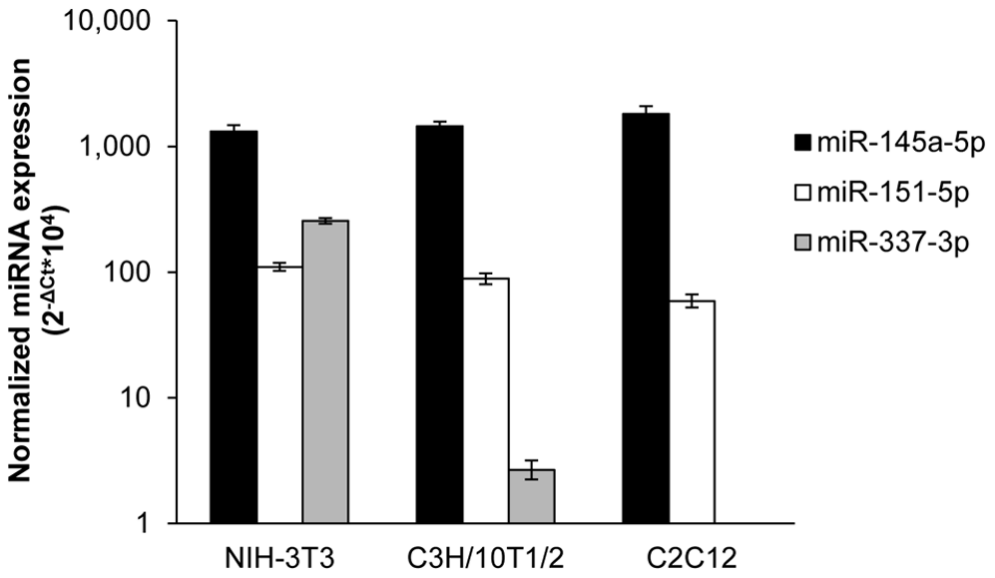
MicroRNA expression levels in different mouse cell lines. Endogenous miRNA expression levels in NIH-3T3, C3H/10T1/2 and C_2_C_12_ cells were measured by qPCR using TaqMan probes, and normalized to U6 snRNA. Due to the high expression of miR-145a-5p, the relative values are presented on a logarithmic scale.

To test whether endogenous miRNAs are able to target the murine *Twist1* 3′UTR reporter, we analyzed the expression pattern of miRNAs of interest in some commonly used mouse cell lines: NIH-3T3, C3H/10T1/2 and C_2_C_12_ (Fig. 5). Since miR-145a-5p, miR-151-5p and miR-337-3p all were expressed in NIH-3T3 and C3H/10T1/2 but not in C_2_C_12_ cells, we selected the first two lines for further analysis.

**Figure 6:**
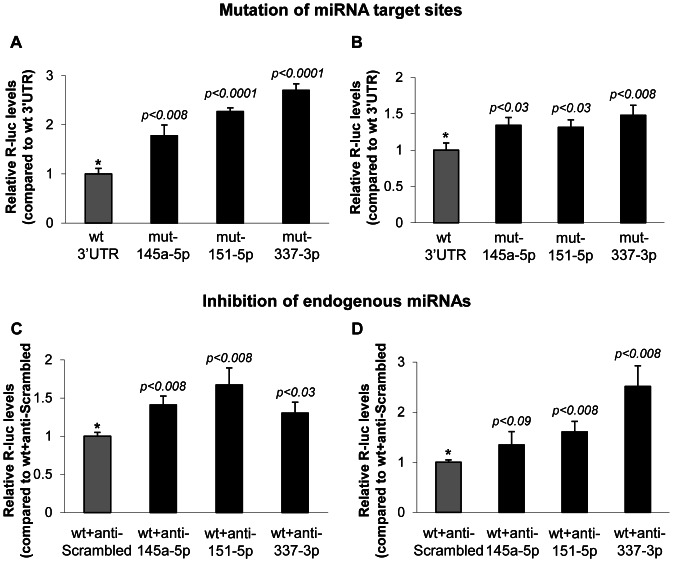
Endogenous microRNAs reduce the activity of *Twist1* 3′UTR reporter. NIH-3T3 (A) and C3H/10T1/2 (B) cells were transfected with wt or mutant psiCheck2-Twist1-3′UTR reporter only. Endogenous miRNAs in NIH-3T3 (C) and C3H/10T1/2 (D) cells were inhibited by co-transfection of 50 nM miRCURY LNA microRNA inhibitors and wt psiCheck2-Twist1-3′UTR reporter. Firefly luciferase activities were measured after 48 h, normalized to *Renilla* luciferase activities and subsequently to the wt 3′UTR (A-B) or wt+anti-Scrambled (C-D). Statistical significance was calculated by using Student's t-test and by comparing the mutant *Twist1* 3′UTR reporters or miRNA specific antagonists with the wt 3′UTR reporter or a scrambled control (*), respectively.

We then analyzed the effect of endogenous miRNAs on the wt and mutant *Twist1* 3′UTR reporters. As shown in Fig. 6A and 6B, endogenous miR-145a-5p, miR-151-5p and miR-337-3p are targeting *Twist1* 3′UTR in both cell lines, as mutating their target sites led to a statistically significant increase of reporter activities. This was confirmed by using locked nucleic acid (LNA)-modified miRNA antagonists to block each of these endogenous miRNAs. Inhibition of miR-145a-5p, miR-151-5p and miR-337-3p resulted in a decreased repression, i.e. an elevated expression of the reporter protein in both cell lines (Fig. 6C and 6D), confirming that the endogenous murine miRNAs are able to target the *Twist1* 3′UTR.

### MiR-151-5p and miR-337-3p reduce the mobility of murine embryonic fibroblast cells

**Figure 7:**
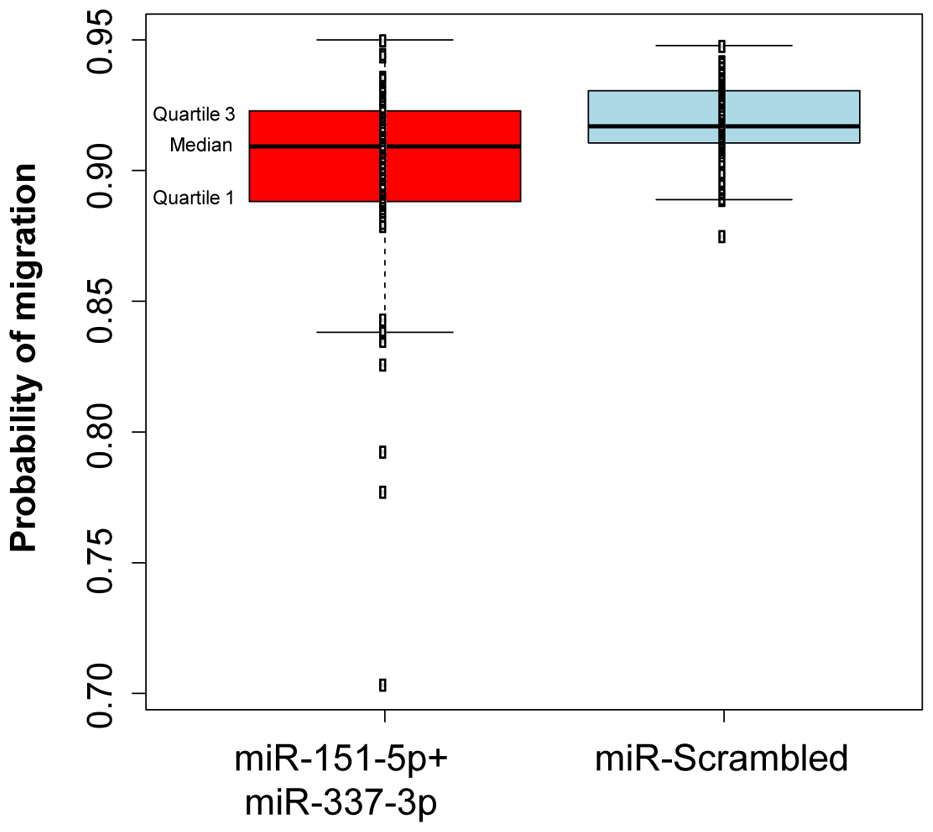
MicroRNAs 151-5p and 337-3p reduce the mobility of murine embryonic fibroblast cells. Dot-plot superposed with the box-plot of the predicted probabilities of cell migration for observations from miR-151-5p + miR-337-3p (red) and scrambled control (blue) treated cells (*p*-value 0.000135). Note the presence of miR-151-5p + miR-337-3p treated cells with extreme low estimates of probability of cell migration.

There is no known cellular function which depends exclusively on TWIST1 and thus would give an all-or-none phenotype upon removal of TWIST1. We therefore decided to test whether the combination of miR-151-5p and miR-337-3p also effects the biological function of endogenous TWIST1, by investigating the ability of murine embryonic fibroblasts to migrate through an 8 µm pore filter. This type of assay correlates well with the EMT promotion by TWIST1 and has been used in similar studies [25]. Compared to cells treated with a scrambled control miRNA, cells treated with a combination of miR-151-5p and miR-337-3p migrated significantly less (Fig.7). Despite the small absolute difference, which probably indicates the limited contribution of TWIST1 to the mobility of these cells, the effect was highly significant. The probabilities of cell migration were estimated as 0.898 (95% bootstrap CI 0.896–0.913) for the miR-151-5p + miR-337-3p treated cells and 0.918 (95% bootstrap CI 0.914–0.922) for the control treated cells, and a bootstrap permutation test (*p*-value 0.000135) showed that the difference between the probabilities is statistically significant.

## Discussion

In this study, we report a new mechanism for the regulation of TWIST1 expression. TWIST1 is a key player in tumorigenesis and metastasis and its overexpression has mostly been correlated with progressed stages of cancer and drug resistance [33]. Furthermore, suppression of *TWIST1* expression in tumor cells can lead to inhibition of metastatic potential [13].

We analyzed the presence of conserved miRNA target sites in the 3′UTR of *Twist1* in selected amniotes (Table 1 and 2) and identified miR-145a-5p, miR-151-5p and the combinations of miR-145a-5p + miR-151-5p and miR-151-5p + miR-337-3p as the strongest regulators of murine *Twist1* (Fig. 2 and 3). For the initial screen, we used human H1299 cells, which are easy to transfect. It should be noted, however, that a strong expression of endogenous miRNAs in these cells might cover the effect of exogenous miRNAs tested and thus give false negative results. In fact, we assume that the relatively high expression of endogenous miR-151-5p in H1299 cells (publically available miRNA microarray dataset; GEO accession number GSE30075) explains the significant difference between the miR-151-5p-mediated repressive effects shown in figures 2 and 3.

The distance between miR-145a-5p and miR-151-5p target sites is 394 nt, too long to expect a synergistic interaction [34]. Indeed, the effect of their combination was only slightly stronger than the additive effect of their independent action. In contrast, the combinatorial effect of miR-151-5p and miR-337-3p clearly is synergistic, as miR-337-3p alone was not able to downregulate the *Twist1* 3′UTR reporter but only did so when miR-151-5p was present (Fig. 3). Importantly, this synergistic effect was not observed if the miR-151-5p target site was mutated. The 6 nt distance between these two miRNA target sites is within the optimal range for two cooperating miRNAs (between 6 to 40 nt) [34]. In addition, miR-337-3p has no complementary sequence to *Twist1* 5′ of its seed region leaving this sequence free for miR-151-5p to bind. Interestingly, the target sites for miR-151-5p and miR-337-3p will still be present, even if the previously reported shortening of 3′UTRs in cancer cells [35] also occurs with *TWIST1* and either the first or the second pA signal should be used.

By mutating miRNA target sites and inhibiting miRNA binding using specific LNA-modified oligonucleotides, we confirmed the effect of the above described miRNAs on the *Twist1* 3′UTR (Fig. 6) and demonstrated that the physiological expression levels of endogenous miRNAs are sufficient for a significant repression of *Twist1* which most likely is due to translational inhibition rather than degradation of *Twist1* mRNA (Fig. 4B). MiR-337 always required miR-151 to be present in order to have an effect. While this work was under revision, miR-214 [36], miR-300, miR-539 and miR-543 [37] have also been reported to target the *TWIST1* 3′UTR. While two of them, miR-539 and miR-300 have no target site in the murine *Twist1* 3′UTR, a third, miR-543 did not show a significant effect in our screen. This might be due to quantitative differences between the transfection of a miRNA expression vector and application of miRNA-mimics. It would be interesting to see whether these miRNAs also target *TWIST1* by inhibiting translation or by RNA destabilization.

All of the miRNAs shown to repress TWIST1 expression have been demonstrated to be involved both in embryonic development and cancer.

During development, miR-145a-5p functions as a critical switch in promoting smooth muscle differentiation and its downregulation is associated with proliferation [38]. MiR-145a-5p is a known tumor suppressor with a strong inhibitory effect on cancer cell proliferation that is downregulated in a variety of tumors including bladder, breast, colon, colorectal, gastric, lung, oral, ovarian, and prostate cancers, hepatocellular and nasopharyngeal carcinoma and pituitary tumors [39], all of which are associated with increased levels of *TWIST1.* In cervical cancer cells, miR-145a-5p was shown to inhibit growth, invasion and therapy resistance [40], problems often observed in tumors in which *TWIST1* is upregulated, and ectopic delivery into mouse pancreatic xenograft models resulted in the inhibition of tumor growth [41], [42].

MiR-151 targets the thrombopoietin receptor MPL [43], a gene known to be downregulated in myeloproliferative neoplasms [44]. In hepatocellular carcinoma (HCC), miR-151-5p has been correlated with cell migration and intrahepatic metastasis [45]. However, miR-151 has been shown to be both upregulated (HCC, nasopharyngeal carcinoma) and downregulated (pappillary carcinoma, acute myeloid leukemia) in different types of cancer [46]. This dual function probably reflects the differences in gene regulation that leads to the various types of cancer. It would thus be interesting to investigate whether the expression of miR-151 in tumors correlates with the expression level of TWIST1.

MiR-337 is upregulated during bone formation [47] whereas TWIST1 is downregulated [48] and overexpression of *Twist1* is known to inhibit osteoblast differentiation [49]. This suggests miR-337 as a potential inhibitory factor regulating *Twist1* during bone formation. In cancer, miR-337 has been shown to be overexpressed in older melanoma patients compared to younger ones [50] and is negatively associated with survival in advanced ovarian cancer [51]. So far, nothing is known about the co-expression of miR-151 and miR-337. In light of our results, it would be interesting to see whether miR-337 is able to acquire a tumor suppressor function when co-expressed with miR-151 in TWIST1 positive tumors.

TWIST1 itself is also known to regulate the expression of several miRNAs, many of which are cancer-related. E.g. the oncogenic miR-10b is directly activated by TWIST1 and promotes the invasion and metastasis through repressing *HOXD10* expression [3]. Also, the miR-200 family and miR-205 were shown to be repressed by TWIST1 [52]. These miRNAs are frequently silenced in advanced cancer [53], [54]. They target ZEB1 and ZEB2, transcriptional repressors of E-cadherin, and thereby inhibit EMT and tumor invasion [55]. Recently, miR-223, overexpressed in metastatic gastric cancer cells, was also found to be induced by TWIST1 [56]. Considering its repression by miRNAs as demonstrated in this study, *TWIST1* appears as a central player in the regulating network of miRNAs and transcription factors necessary for cellular homeostasis.

Recently, RNAi-mediated silencing of TWIST1 was shown to suppress the proliferation of human cervical cancer cells and to improve the chemosensitivity to cisplatin treatment, indicating a novel therapeutic strategy to overcome drug resistance [57]. Likewise, Burns *et al*. demonstrated a strong therapeutic effect in oncogene driven non-small cell lung cancer after silencing TWIST1 expression [58]. Our results about the miRNA regulation of TWIST1 provide an alternative approach to suppress this potent oncogene utilizing an endogenous mechanism.

The reduced mobility of miR-151-5p + miR-337-3p treated embryonic fibroblasts further shows the therapeutic potential in the combination of these two miRNAs (Fig. 7). The relatively small absolute difference in the migration probability is most likely explained by the fact that cell motility is a property influenced by a number of different factors. However, the high statistical significance of the difference indicates that the treatment with miR-151-5p and miR-337-3p has a reliable effect.

In summary, we have shown that TWIST1 is regulated by miR-145a-5p, miR-151-5p and miR-337-3p. The additive and synergistic effects of these miRNAs could reduce unwanted ‘off target’ effects and might open up new possibilities to specifically interfere with *TWIST1* translation in therapeutic approaches.

## Materials and Methods

### Sequence comparison

Sequence comparison was done with CLC Main Workbench Version 5.7 (CLC bio). Parameters for alignments were: gap open cost  = 10 and gap extension cost  = 1. The following sequences were pairwise compared: *Twist1*: human (GeneBank: NM_00047); cow (GeneBank: XM_002686684); mouse (GeneBank: NM_011658); chicken (GeneBank: NM_204739; 3′UTR without nt 845-873 and 899-971 which correspond to 2 stretches of sequence not found in any other species). *Twist2*: human (GeneBank: NM_057179); cow (GeneBank: NM_001083748); mouse (GeneBank: NM_007855); chicken (GeneBank: NM_204679). In addition, *Twist1* sequences used for miRNA target site analysis were: dog (GeneBank: XM_857736); frog (GeneBank: NM_001085883).

### Cell lines and constructs

All cell lines were obtained from American Type Culture Collection and cultured at 37°C in 5% CO_2_. H1299 cells were maintained in RPMI 1640 (Gibco) and NIH-3T3, C3H/10T1/2 and C_2_C_12_ cells in DMEM (Gibco), all supplemented with 10% FCS, 100 µg/ml streptomycin and 100 U/ml penicillin (Gibco).

Murine pre-miRNA sequences with about 200 nt 5′ and 3′ flanking regions were amplified from C57Bl/6J mouse genomic DNA and cloned into NotI/SalI sites in the pdCMV2-EGFP expression vector. The miRBase accession numbers for miRNAs are: mmu-miR-33 (MI0000707), mmu-miR-145a (MI0000169), mmu-miR-151 (MI0000173), mmu-miR-326 (MI0000598), mmu-miR-337 (MI0000615), mmu-miR-361 (MI0000761), mmu-miR-378a (MI0000795), mmu-miR-381 (MI0000798), mmu-miR-409 (MI0001160) and mmu-miR-543 (MI0003519). Accession numbers for two miRNAs used as negative controls are: hsa-miR-485 (MI0002469) and hsa-miR-609 (MI0003622). Primers used for cloning were: mmu-miR-33-upper (5′ctgtcagcggccggaagcctactcgggcaatgtgcc 3′), mmu-miR-33-lower (5′ gctgcgatcgtcgacggaagcagctagttctaacctcc 3′), mmu-miR-145a-upper (5′ agctatgcggccggactctacgcacatgaaatgcttcttcc 3′), mmu-miR-145a-lower (5′ gctgcgatcgtcgacggattggatgtaatataaatgaagcaaacc 3′), mmu-miR-151-upper (5′ ctgtcagcggccgctttagggcctgagaatatcttga 3′), mmu-miR-151-lower (5′ gggaccgcgtcgacgaagcaaacctcaataacagaactc 3′), mmu-miR-326-upper (5′ cctgtagcggccgctactccgcatagccactgaga 3′), mmu-miR-326-lower (5′ gcggatccgtcgacctagcccagggccatatacatg 3′), mmu-miR-337-upper (5′ ctgtcagcggccgccatgagagtttataagaagtgagg 3′), mmu-miR-337-lower (5′ gggaccgcgtcgacgccaaggaatgatctcaggtatgg 3′), mmu-miR-361-upper (5′ agctatgcggccgcttaacatgccttggtttgcaga3′), mmu-miR-361-lower (5′ acctagcggtcgacctttgctgctttctttgcttcc 3′), mmu-miR-378a-upper (5′ cctgtagcggccggtaattgataccaggagctttgcagcc 3′), mmu-miR-378a-lower (5′ acctagcggtcgacccagacagctcacatgcaaacacagg 3′), mmu-miR-381-upper (5′ catgtagcggccgccaatctgttgtaacatctgccagt 3′), mmu-miR-381-lower (5′ gctgcgatcgtcgacagaacatgcacacttgggtac 3′), mmu-miR-409-upper (5′ cctgtagcggccggggaaggctttagatattcttggaagg 3′), mmu-miR-409-lower (5′ acctagcggtcgaccacggtcgatctcccttcaagtaccagc 3′), mmu-miR-543-upper (5′ agctatgcggccgggagactccaaagacctccccaaagg 3′) and mmu-miR-543-lower (5′ gggaccgcgtcgacgtggaggagggaggagggagcaggagcc 3′).

Mouse *Twist1* 3′UTR sequence (GeneBank: NC_000078; genomic DNA position 34,643,544 to 34,645,282) was cloned into the XbaI/NotI sites of pGL3-control vector (Promega). This sequence includes the unique intron of the *Twist1* gene and thus represents a construct which will report all aspects of *Twist1* mRNA processing including splicing. PRluc-N2 (PerkinElmer) encoding *Renilla* luciferase was used for normalization.

Due to high fluctuations in the pGL3-control transfection efficiencies, mouse *Twist1* 3′UTR sequence (GeneBank: NM_011658; nucleotide position 926-1634) was cloned into XhoI/NotI sites in psiCheck2 vector (Promega) which expresses both Firefly and *Renilla* luciferases. MiRNA target sites were subsequently mutated to restriction enzyme recognition sites: miR-145a-5p to SacI; miR-151-5p to AgeI; miR-337-3p to SalI.

Stable pools of cells expressing psiCheck2-Twist1-3′UTR were established as described in [59].

### Transfections and luciferase assays

All transfections were carried out in triplicates on 48-well plates in three independent experiments and assayed after 48 h using the Dual-Luciferase Reporter Assay System Kit (Promega). 48 h was chosen to allow the cells to recover from the transfection and grow up to ∼80–100% confluence at the time of measurement.

In the miRNA screen, 10^4^/cm^2^ H1299 cells were transfected with 250 ng pdCMV2-EGFP-miR-X (or 125 ng each if two miRNAs were combined), 50 ng pRluc-N2 and 50 ng pGL3-Twist1-3′UTR or pGL3-control using TransIT-LT1 transfection reagent (Mirus) according to manufacturer's guidelines. For normalization, the Firefly luciferase activity was divided by the *Renilla* luciferase activity. Triplicates were averaged and normalized to the pGL3-control.

In the target site mutation assay, 10^4^/cm^2^ H1299 cells were transfected with 250 ng (or 125 ng each) miRNA construct and 50 ng wt or mutant psiCheck2-Twist1-3′UTR using TransIT-LT1 transfection reagent (Mirus) according to manufacturer's instructions. For normalization, *Renilla* luciferase activity was divided by Firefly luciferase activity and the mutant-transfected cells were subsequently normalized to the respective wt 3′UTR reporter treated with miRNA.

The transfection efficiency of miRNA-encoding vectors was monitored by the expression of EGFP with the Leica DM fluorescence microscope. The efficiency was always above 60%.

10^4^/cm^2^ NIH-3T3 and C3H/10T1/2 cells were transfected with 50 ng psiCheck2-Twist1-3′UTR using Fugene HD transfection reagent (Roche) according to manufacturer's guidelines. 50 nM miRCURY LNA microRNA inhibitors (Exiqon) were added in the inhibition assay: mmu-miR-145a-5p (139465-00), hsa-miR-151-5p (414654-00), mmu-miR-337-3p (139530-00) and a Scrambled negative control (199004-00). For normalization, *Renilla* luciferase activity was divided by Firefly luciferase activity.

10,000 stable psiCheck2-Twist1-3′UTR-expressing cells per 96-well plate well were transfected while seeding in 100 µl serum-free RPMI 1640 (Gibco) using 0.2 µl Lipofectamine 2000 (Invitrogen) and 20 or 5 nM Pre-miR miRNA Precursor (Applied Biosystems) per well: pre-mmu-miR-151 (PM11537), pre-mmu-miR-337 (PM12817) and Scrambled Negative Control #1 (AM17110). Simultaneously, 30,000 cells/well were transfected on a 24-well plate for RNA extraction and analysis using more reagents, accordingly. After 4 h of transfection, 10% FCS (Gibco) of total volume was added to each well. *Renilla* luciferase mRNA and protein levels were measured after 48 h.

### Quantitative PCR

Total RNA was purified using TRIzol (Invitrogen) according to the manufacturer's instructions. 500 ng of total RNA was reverse transcribed with M-MLV reverse transcriptase (Invitrogen) using 200 ng random hexamer primer (Roche).

*Renilla* luciferase expression was quantified using Platinum SYBR Green qPCR SuperMix-UDG (Invitrogen) and 25 ng cDNA as template. The primers used were: R-luc_fwd (5′ gttccctaacaccgagttcgtg 3′), R-luc_rev (5′ ctccacgaagctcttgatgtac 3′), F-luc_fwd (5′ ctgatcgacaaggacggctgg 3′), F-luc_rev (5′ ggtagcccttgtacttgatcag 3′). *Renilla* luciferase mRNA levels were normalized to Firefly luciferase mRNA levels and subsequently to cells transfected with the negative control. Relative quantification of mRNA levels was performed using the ΔΔCt-method.

For miRNA analysis, 25 ng of total RNA was reverse transcribed using TaqMan MicroRNA Reverse Transcription Kit (Applied Biosystems). QPCR was carried out in triplicates using 1.33 µl cDNA and TaqMan Universal PCR Master Mix (Applied Biosystems). The TaqMan MicroRNA Assays used were: hsa-miR-145a-5p (002278), hsa-miR-151-5p (002642), mmu-miR-337-3p (002532) and U6 snRNA (001973). Relative quantities of miRNA were calculated by the ΔCt method.

### Cell migration assay

3T3 immortalized murine embryonic fibroblasts [60] were plated on a 12-well plate at a density of 10^4^/cm^2^ in 1 ml complete growth medium. While seeding, cells were transiently transfected with a total of 20 nM pre-mmu-miR-151-5p (PM11537) and pre-mmu-miR-337-3p (PM12817) or with Scrambled Negative Control #1 (AM17110; all Applied Biosystems) using Lipofectamine 2000 (Invitrogen) according to manufacturer's instructions. After 24 h, cells were trypsinized, resuspended in 400 µl medium, distributed equally on 2 cell culture inserts 200 µl each (24-well, 8.0 µm pore size, Falcon, 35 3097) and then placed in a 24-well plate (Falcon, 35 3504) which contained 600 µl medium per well. The 2 inserts were designated as ‘A,over’ and ‘B,under’ to study the analysis of non-migrated and migrated cells, respectively. Cells were allowed to migrate for 21 h, washed with PBS and fixed in 4% freshly dissolved paraformaldehyde for 30 min at room temperature. Following 2× washing with PBS, the cells on the lower surface of insert A and the cells on the upper surface of insert B were wiped off with a cotton swap. The insert membranes were cut off and embedded in ProLong Gold Antifade Reagent with DAPI (Invitrogen). Migrated (under) and non-migrated (over) cells were counted in 10 (primary magnification 10×) and 5 (primary magnification 5×) random areas, respectively, on micrographs taken with a Leica DM fluorescence microscope.

### Statistical analysis

Statistical significance for the transfection experiments was calculated using Student's t-test and 2-sided *p*-values.

The effect of the treatment on the probability of cell migration was studied in the following way: Denote the counts of the migrated cells and the non-migrated cells for the *m* ^th^ observed microscopic field for the *r* ^th^ replication subject to the *t* ^th^ treatment by *M_mrt_* and *S_mrt_*, respectively. The expected number of migrated and non-migrated cells are given by E(*M_mrt_*) = *p_t_ U_rt_* and E(*S_mrt_*) = *(1-p_t_) U_rt_/40*, respectively. Here *p_t_* is the probability of migration common to all the observations subject to the *t* ^th^ treatment and *U_rt_* is the expected number of cells transferred from the suspension associated to the *rt* ^th^ replicate. The factor 1/40 arises from the fact that different macroscopic field areas were observed for the independent determinations of the migrated and non-migrated cells. Using a first order Taylor expansion yields that the expectation of *P*_t = *Mmrt*_/(*M_mrt_* + *S_mrt_*) is the probability *p_t_*, therefore *P*_t_ is an unbiased estimate of *p_t_*. The effect of the treatment on the probability of cell migration was then formally tested using the Monte Carlo permutation test [61] with 1,000,000 bootstrap permutations. Moreover, 95% bootstrap confidence intervals for the probabilities of cell migration were constructed with non-parametric bootstrap [61], with 10,000 parametric bootstrap each.
